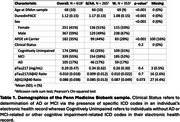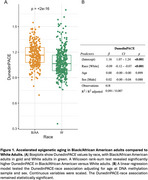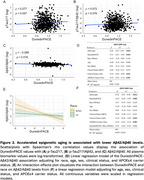# Epigenetic age acceleration is faster in Black adults and is associated with plasma amyloid

**DOI:** 10.1002/alz70855_107267

**Published:** 2025-12-25

**Authors:** Rory Boyle, Hao Xu, Katheryn A. Q. Cousins, Nadia Dehghani, Colleen Morse, Anurag Verma, Leslie M. Shaw, Lauren Massimo, Wanding Zhou, David A. Wolk, Corey T. McMillan, Penn Medicine Biobank

**Affiliations:** ^1^ Department of Neurology, University of Pennsylvania Perelman School of Medicine, Philadelphia, PA, USA; ^2^ University of Pennsylvania, Philadelphia, PA, USA; ^3^ Children's Hospital of Philadelphia, Philadelphia, PA, USA

## Abstract

**Background:**

We have previously reported accelerated epigenetic aging in Black individuals relative to White counterparts in a population‐based cohort which further associates with increased cognitive impairment and decline. However, the extent to which this disparity relates to AD in unclear. In a diverse and real‐world clinical dataset, we examined whether there are disparities in accelerated epigenetic aging across self‐identified Black and White adults and the extent to which it associates with AD plasma biomarkers.

**Method:**

618 Participants aged 50+ from the electronic health record (EHR)‐linked Penn Medicine Biobank had available data for blood‐based DNA methylation and plasma biomarkers of AD (Table 1). International Classification of Disease codes determined clinical status (AD, MCI, and cognitively unimpaired). DNA methylation data were processed using the Illumina Methylation Screening Array and DunedinPACE estimates of epigenetic aging were obtained using the dnaMethyAge R package. Plasma analytes (*p*‐tau217, *p*‐tau217/Aβ42, Aβ42/Aβ40) were assayed using Fujirebio Lumipulse G1200.

**Result:**

Black adults had higher epigenetic estimates of DunedinPACE consistent with accelerated aging (Figure 1A) and this association was independent of age at blood draw, and sex (β = ‐0.09, *p* <0.001, Figure 1B). DunedinPACE had positive, but non‐significant, associations with *p*‐tau217 (rho=0.077, *p* = 0.057, Figure 2A) and *p*‐tau217/Aβ42 (rho=0.072, *p* = 0.076, Figure 2B). DunedinPACE was negatively associated with Aβ42/Aβ40 (rho=‐0.099, *p* = 0.016, Figure 2C), indicating greater amyloid burden with accelerated aging, and this association was significant when adjusting for chronological age, sex, race, APOE‐ε4 status, and clinical status in a linear regression (β=‐0.11, *p* = 0.024, Figure 2D). A marginal interaction between DunedinPACE and race suggests that this association may be marginally stronger in Black adults compared to white adults (β=0.19, *p* = 0.062, Figs. 2E‐F).

**Conclusion:**

We observed that epigenetic aging, measured with DunedinPACE, is accelerated in Black relative to White adults, associated with lower Aβ42/Aβ40 levels, and that this association may be stronger in Black adults. An epigenetic clock trained on a representative dataset is needed to provide better generalizability and to accurately determine the association of epigenetic aging with AD biomarkers in Black adults.